# Supplementary material for: Content-rich biological network constructed by mining PubMed abstracts
Source: BMC Bioinformatics. 2004 Oct 8;5:147. doi: 10.1186/1471-2105-5-147 (PMC528731; doi:10.1186/1471-2105-5-147)
Supplement: Additional File 2 — The original results of the above study (non-essential files are deleted to keep the file size under the limit set by BMC bioinformatics). [file 1471-2105-5-147-S2.bz2 › chilibotAdditionalFile2/dip10/538ID11085509E10890/html/AR_BRCA1.html]

 


 **AR** and **BRCA1** 
  
Found 28 abstracts in PubMed, retrieved 10.  
 

 What does Google say? 
 PDF only 
| .edu only 

---

**Interactive relationship** (e.g. stimulation, inhibition, etc)

**Neutral relationship**- RESULTS The  **AR**  was only present in 12% of  **BRCA1**  mutated tumors, with mutations located at the C terminal half of the  **BRCA1**  gene.  Ref: 12779088 Breast Cancer Res Treat, 2003
- The CAG repeat within exon 1 of the androgen receptor  [ **AR** ]   **AR**  gene is reportedly associated with breast cancer phenotype in  **BRCA1**  mutation carriers.  Ref: 12404104 Eur J Hum Genet, 2002
- Androgen receptor  [ **AR** ]  CAG repeat length in Jewish Israeli women who are  **BRCA1**  2 mutation carriers association with breast ovarian cancer phenotype.  Ref: 12404104 Eur J Hum Genet, 2002
- Two hundred and twenty seven  **BRCA1**  2 mutation carriers were genotyped for the polymorphic  **AR**  CAG repeat, and allele size was correlated with breast ovarian cancer morbidity parameters.  Ref: 12404104 Eur J Hum Genet, 2002
- The loss of the  **AR**  expression, as shown by immunohistochemistry, together with the observed loss of other steroid hormone receptors in  **BRCA1**  mutated tumors may lead to a hormone independent growth or to anti hormone resistant growth of these tumors.  Ref: 12779088 Breast Cancer Res Treat, 2003

**Non-interactive relationship** (e.g. studied together, co-existance, homology, etc.)

- Since this growth factor is induced by androgens, we studied the androgen receptor  [ **AR** ]   **AR**  expression in BRCA  [ **BRCA1** ]  mutated tumors and in matched sporadic breast tumors.  Ref: 12779088 Breast Cancer Res Treat, 2003
- METHODS Paraffin embedded breast tumors of carriers of a  **BRCA1**  mutation n=41, median age of patients at time of surgery was 41 years range 28 59 years or a BRCA2 mutation n=14, median age 41 years range 31 85 years were analyzed for the presence of ER alpha, PR, P53 and  **AR**  using standard immunohistochemical techniques.  Ref: 12779088 Breast Cancer Res Treat, 2003
- There is no conclusive evidence of association between  **AR**  CAG repeat size and breast or ovarian cancer risk in Jewish  **BRCA1**  2 mutation carriers.  Ref: 12404104 Eur J Hum Genet, 2002
- We have extended our LOH analysis of chromosome 3p to include 102 ovarian tumor specimens 29 and 73 samples were previously examined for LOH of chromosome 3p and 17 markers, respectively, using additional polymorphic markers, to assess the coordinate LOH of loci representing the three chromosome 3p minimal regions of deletions von Hippel Lindau syndrome VHL, thyroid hormone receptor beta, and fragile histidine triad FHIT and LOH of other important loci tumor protein 53 TP53, breast cancer 1 early onset  [ **BRCA1** ]   **BRCA1** , breast cancer 2 early onset, retinoblastoma 1, ornithine carbamoyltransferase, and androgen receptor  [ **AR** ]  or somatic mutations in TP53.  Ref: 12112314 Mol Carcinog, 2002
- BRCA2 not only associates with  **AR**  and GRIP1 but also cooperates with both the histone acetyltransferase P CAF and  **BRCA1**  to enhance  **AR**  and GRIP1 mediated transactivation.  Ref: 12756300 Proc Natl Acad Sci U S A< MedlineTA>Proc Natl Acad Sci U S A, 2002
- No effect on survival was seen for polymorphisms in ATM,  **BRCA1**  2, CHK2, KU70, NBS1, RAD51, RAD52, XRCC3,  **AR** , COMT, NQO1, VDR, ADH3, CYP1A1, GSTP1, TGF beta, or CDH1.  Ref: 12036913 Cancer Res, 2002
